# Barbigerone attenuates 3-nitropropionic acid-induced Huntington’s disease-like neuropathology in rats via antioxidant, anti-inflammatory, and neuroprotective mechanisms

**DOI:** 10.1038/s41598-025-07181-5

**Published:** 2025-07-02

**Authors:** Sattam Khulaif Alenezi

**Affiliations:** Department of Pharmacology and Toxicology, College of Pharmacy, Qassim University, Buraydah, 51452 Al Qassim Saudi Arabia

**Keywords:** 3-Nitropropionic acid, Barbigerone, Cognitive function, Huntington’s disease, Oxidative stress, Neuroprotective, Motor deficits, Neuroscience, Cognitive neuroscience

## Abstract

Huntington’s Disease (HD), a neurodegenerative disease characterized by motor and cognitive impairments, arises from genetic mutations causing protein aggregation within the brain. The 3-Nitropropionic acid (3-NPA) rat model mimics key features of HD. This study explored the therapeutic efficacy of barbigerone, a compound with antioxidant and anti-inflammatory properties, in ameliorating 3-NPA-induced neurodegeneration and cognitive deficits in rats. Male Wistar rats were randomized into four groups: a normal control group, a 3-NPA control group, and two groups treated with different doses of barbigerone along with 3-NPA. Behavioral test, biochemical assays, and histopathological examinations were performed. Barbigerone significantly (*P* < 0.0001) restored motor coordination, grip strength, and mobility compared to 3-NPA-induced HD rats. Barbigerone concomitantly reduced oxidative stress by lowering malondialdehyde (MDA) and nitric oxide (NO) levels, while enhancing antioxidant enzymes such as glutathione (GSH), superoxide dismutase (SOD), and catalase (CAT). Furthermore, treatment modulated the pro-inflammatory cytokines, suggesting a reduction in neuroinflammation. Barbigerone significantly (*P* < 0.0001) impacted changes in acetylcholinesterase (AChE) activity and modulated levels of key neurotransmitters, such as acetylcholine (ACh), norepinephrine (NE), serotonin (5-HT), gamma-aminobutyric acid (GABA), dopamine (DA), and glutamate (GLU). Additionally, barbigerone effectively attenuated the 3-NPA-induced elevation of caspase-3 and caspase-9, while upregulating brain-derived neurotrophic factor (BDNF), which is crucial for neuronal survival and cognitive function. Histopathological examination revealed that barbigerone significantly restored the altered striatal architecture, indicating a protective effect against neurodegeneration. Barbigerone exhibits potential as a therapeutic choice for HD, offering the possibility of alleviating motor impairments, oxidative stress, inflammation, and cognitive dysfunction.

## Introduction

Huntington’s disease (HD) is an inherited autosomal dominant neurodegenerative disorder characterized by progressive motor dysfunction, cognitive decline, and psychiatric disturbances, primarily due to expanded cytosine-adenine-guanine (CAG) repeats in the huntingtin (HTT) gene, which result in the production and aggregation of mutant huntingtin (mHTT) protein in neurons^1,2^. These protein aggregates trigger a cascade of neurotoxic events, including mitochondrial dysfunction, oxidative stress, excitotoxicity, and neuroinflammation^3^. The disease predominantly affects gamma-aminobutyric acid (GABA)-producing medium spiny neurons in the striatum, leading to characteristic motor symptoms such as chorea, dystonia, and rigidity^4^. Clinically, HD presents with a triad of motor, cognitive, and psychiatric abnormalities, with cognitive symptoms typically involving memory impairment and difficulties in thought processing and organization^5,6^.

Multiple mechanisms contribute to HD pathogenesis, including altered neurotransmission, particularly involving dopaminergic, glutamatergic, and GABAergic systems. Reduced GABA levels, along with aberrant dopamine (DA) and serotonin metabolism, have been reported in the striatum of HD patients and animal models, contributing to both motor and cognitive dysfunctions^7,8^. The HTT mutation also affects astrocyte and microglial function, promoting the release of inflammatory cytokines. Elevated levels of IL-6, chitotriosidase, and YKL-40 in cerebrospinal fluid further indicate the involvement of the innate immune system and chronic neuroinflammation^9^.

3-Nitropropionic acid (3-NPA), a mycotoxin derived from fungal and plant sources, is widely used to model HD in experimental animals. Its principal mechanism involves irreversible inhibition of succinate dehydrogenase (SDH), a critical enzyme in the mitochondrial electron transport chain^10^. In both rodents and humans, 3-NPA administration induces a spectrum of motor impairments including dystonia, involuntary movements, and memory deficits. The neurotoxic effects of 3-NPA are multifaceted, encompassing adenosine triphosphate (ATP) depletion, disrupted calcium homeostasis, excitotoxicity, oxidative stress, and ultimately neuronal death^11^. As a suicide inhibitor of SDH, it impairs respiratory electron transfer, resulting in mitochondrial dysfunction. Neurons, especially in the striatum, are particularly vulnerable to this metabolic disruption. Due to its ability to replicate HD-like neuropathology and clinical features, 3-NPA is extensively employed as a neurotoxin in preclinical HD models^12^.

In recent years, flavonoids have gained attention for their neuroprotective potential in treating various neurodegenerative conditions due to their antioxidant, anti-inflammatory, and anti-apoptotic properties^13,14^. Barbigerone, a naturally occurring pyranoisoflavone isolated from the seeds of *Tephrosia barbera* and the herb *Sarcolobus globosus*, has been shown to possess antioxidant, anti-inflammatory, and neurotropic properties^15,16^. Previously reported its ability to modulate neurotransmitter levels, suppress proinflammatory cytokines, and enhance cognitive function, making it a promising candidate for neurodegenerative disease management^17^.

Despite its reported bioactivity, the potential role of barbigerone in the treatment of HD has not yet been elucidated. Therefore, the present study aimed to investigate the therapeutic potential of barbigerone in a 3-NPA-induced rat model of HD. The focus was on evaluating whether barbigerone could ameliorate motor and behavioral deficits, restore neurotransmitter balance, attenuate oxidative stress and inflammation, and protect striatal neurons from degeneration. A combination of behavioral assessments, biochemical assays, and histopathological analyses was employed to elucidate the protective effects and mechanisms of action of barbigerone.

## Methodology

### Animals

Adult male Wistar rats (3–5 months old, 150–180 g) were utilized as an established model for HD induced by 3-NPA. Rats were kept in a controlled environment facility, adhering to standard animal husbandry practices and given *ad libitum* access to water and standard rodent chow. A 10-day acclimatization period preceded the initiation of the study to minimize stress. The rats were subjected to a naive treatment protocol, ensuring minimal handling and disturbance. All experimental protocols were carried out in strict adherence to the ethical guidelines and approved by the Institutional Research Board of Qassim University, Al-Qassim, Saudi Arabia (24-12-05). The study was reported in accordance with the Animal Research: Reporting of In Vivo Experiments (ARRIVE) guidelines^18^. No prior surgical or experimental interventions were performed on the animals prior to the commencement of the experimental study.

### Chemicals

**Fig. 1:**
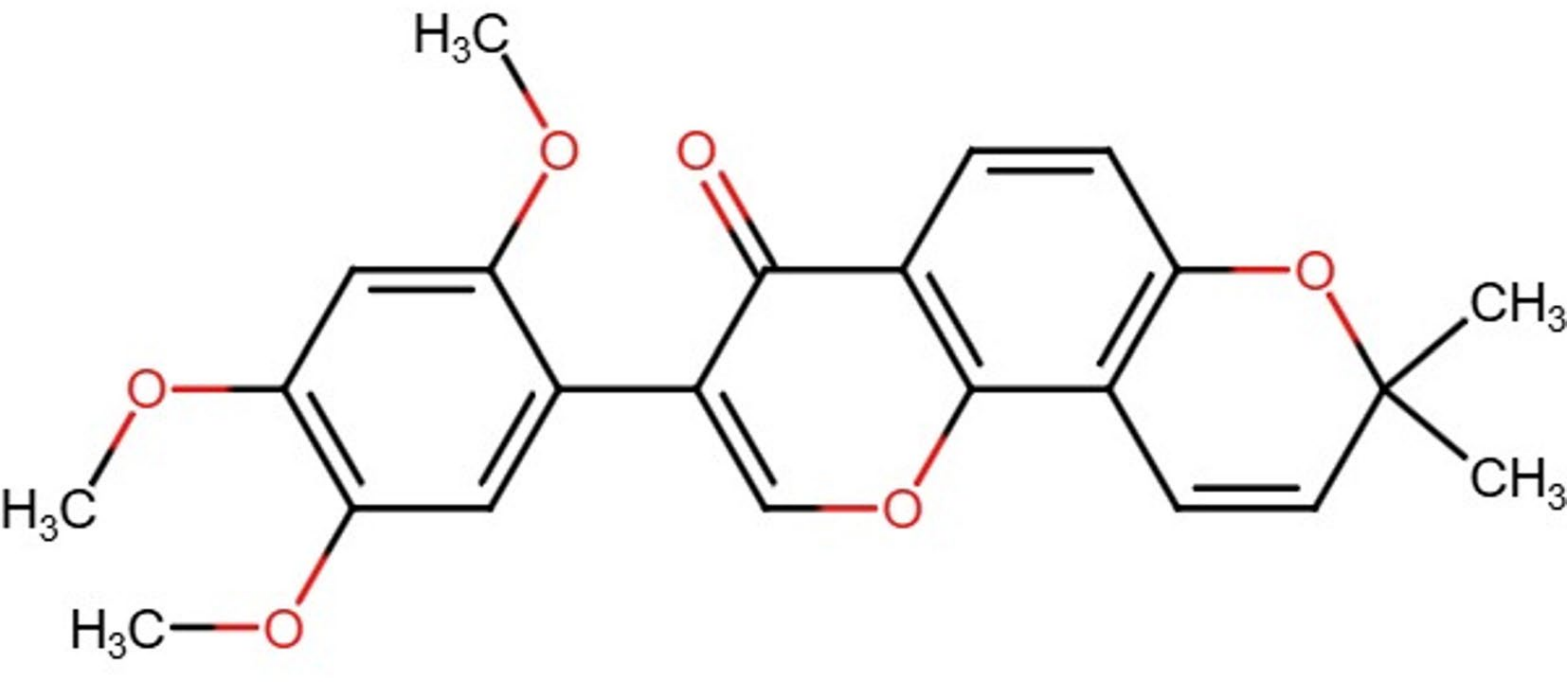
Chemical structure of barbigerone.

The Barbigerone (Fig. 1) and 3-NPA utilized in this investigation were sourced from MSW, Pharma, M.S., India. Biochemical parameters were assessed using commercially available enzyme-linked immunosorbent assay (ELISA) kits. Specifically, kits procured from Krishgen Biosystems, USA, were utilized to quantify pro-inflammatory cytokines, including tumor necrosis factor-alpha (TNF-α, KB3145), interleukin-1 beta (IL-1β, KLR0119), interleukin-6 (IL-6, KB3068), nuclear factor kappa-light-chain-enhancer of activated B cells (NF-κB, KLR0287), caspase-3 (Casp-3, KLR1648), (Casp-9 MSW09, MSW Pharma, M.S., India) and brain-derived neurotrophic factor (BDNF, KLR0476). The analyses conducted in the recent research were carried out using high-quality chemical compounds.

### Experimental design

Intraperitoneal administration of 3-NPA (10 mg/kg) was carried out for 14 consecutive days, following dilution in pH 7.4 saline, to induce alterations in neurotransmitter metabolites and trigger inflammatory responses characteristic of HD^19,20^. The rats were simply randomized into four groups (*n* = 6 per group).


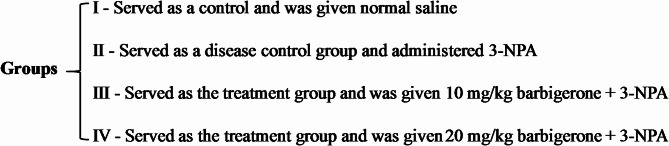


**Fig. 2:**
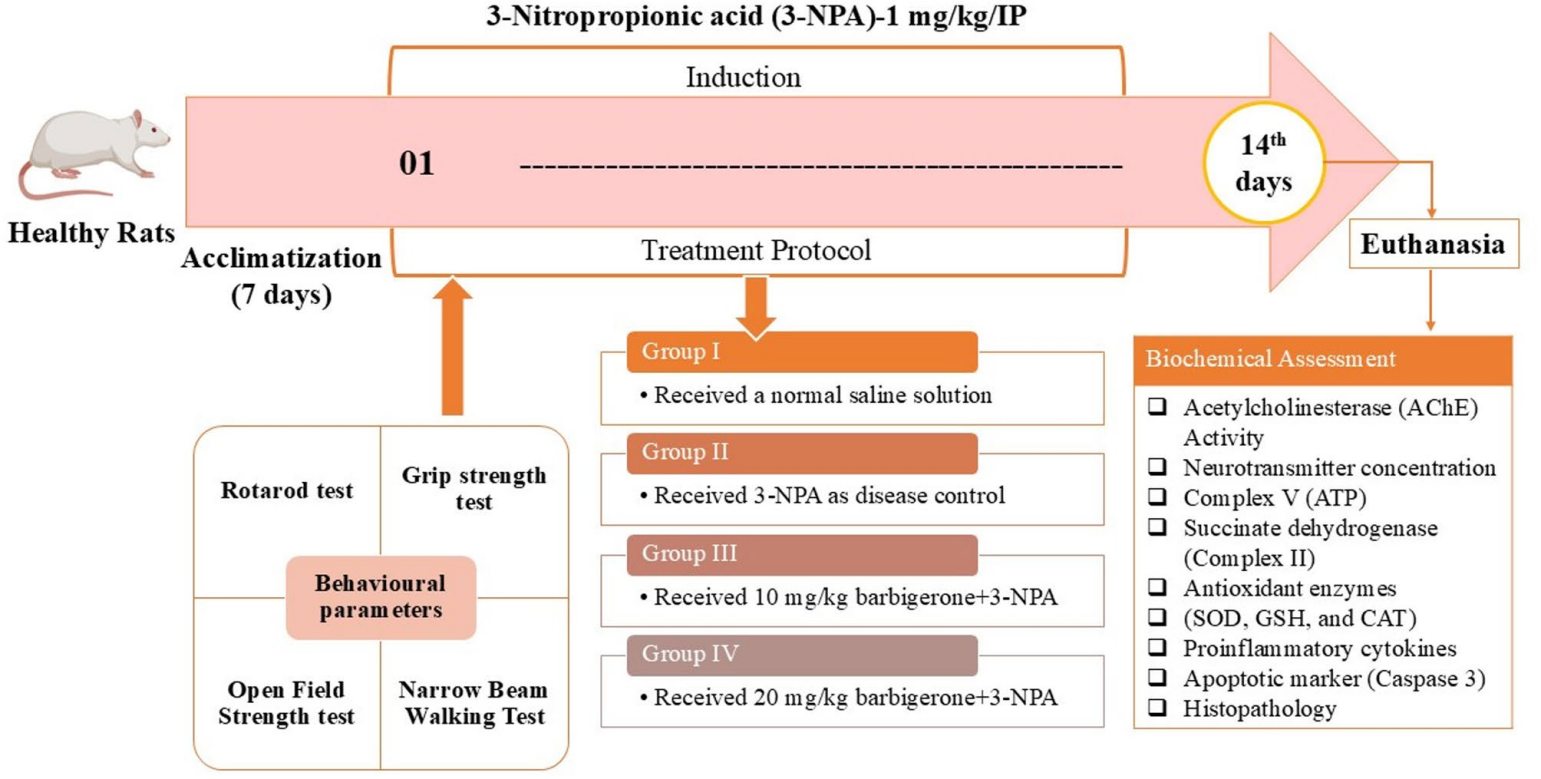
Experiment design.

Behavioral assessments of the experimental animals were performed two hours post-administration of 3-NPA^21^, adhering to their respective treatment groups. Upon completion of the experimental protocol, rats were anesthetized by intraperitoneal injection of ketamine (75 mg/kg) and xylazine (10 mg/kg). Following anesthesia, rats were euthanized by cervical dislocation, and the brain (striatum) was dissected and subjected to centrifugation. The supernatant obtained after centrifugation was then utilized for subsequent biochemical analyses. The experimental design is illustrated in Fig. 2.

### Behavioural parameters

#### Rotarod test

The rotarod test assesses the motor coordination of rats. Each rat underwent a five-day trial period before the actual experiment. Animals capable of remaining on the rotating rod for at least 5 min were selected for the study. The rod used had dimensions of 120 cm in length and 3 cm in diameter, rotating at a speed of 20 revolutions per minute. The latency to fall off the rod was recorded on the test day following the open field study^19^.

#### Grip strength test

A grip strength test was utilized to assess the physical strength of the rats. During the test, each rat was gently held by the tail and allowed to grasp a trapeze bar with its forepaws. The rat was allowed to grip the bar, and the maximum force before release was recorded. To account for the potential influence of body weight on grip strength, all rats were weighed prior to the test. The values obtained during 30 min were recorded, and the mean of these readings was used to determine the animal’s grip power^22^.

#### Open field strength test

A 0.8 m x 0.8 m x 0.4 m cuboid apparatus constructed of wood with red internal walls, a polished black floor, and white gridlines dividing 16 equal squares was employed to evaluate the impulsive locomotor behaviors of the rat. In a sound-isolated room, an overhead camera was positioned to record the rat’s behaviour during the test. Rats were subjected alone in the center of the box for a duration of three minutes to explore the environment independently. Various metrics were recorded during this period, including the distance traveled, mean speed, immobility time, time spent actively in the central squares, and the number of rearing behaviours exhibited by the rats^19^.

#### Narrow beam walking test

Motor function assessment in rodents following hind-limb damage was conducted utilizing a narrow beam walking test. It consists of a long wooden beam (150 cm length × 4 cm width × 3 cm height) elevated 80 cm above the ground, supported by plywood bars at each end. Following a 2-minute acclimation period, animals were assessed for their ability to traverse the beam. The frequency of paw slips and the temporal interval required to traverse the designated beam were meticulously noted. Failure to traverse the beam within a designated timeframe was recorded as the maximum latency. The overall performance metric was determined by calculating the mean of three measured transfer latencies and incorporating an inter-trial interval of 2 min^23^.

### Biochemical parameters

#### Brain tissue homogenization

On day 15, animals were euthanized under light anesthesia via cervical dislocation. Striatal tissues were rapidly dissected from each brain and homogenized in phosphate buffer for biochemical analysis. The homogenates were centrifuged at 5000 rpm for 30 min at 4 °C. The resulting supernatant was collected for the estimation of multiple biochemical markers, including acetylcholinesterase (AChE), DA, norepinephrine (NE), serotonin (5-HT), GABA, glutamate (Glu), malondialdehyde (MDA), nitric oxide (NO), reduced glutathione (GSH), superoxide dismutase (SOD), catalase (CAT), and proinflammatory cytokines (IL-1β, IL-6, TNF-α, NF-κB), as well as apoptotic markers (caspase-3, caspase-9) and BDNF. The crude mitochondrial pellet obtained from the recentrifugation step was used for the analysis of mitochondrial enzymes such as succinate dehydrogenase (SDH) and ATP levels.

#### Acetylcholinesterase (AChE) activity

In accordance with previously documented procedures, acetylcholine (ACh) was utilized as the reagent for assessing AChE activity in both the hippocampus and serum. To initiate the assay, a test mixture consisting of 0.1 M, 2.6 ml of either brain homogenate or plasma, and 0.4 ml was combined with 100 µl of DTNB at a pH of 8.0. Prior to adding the test solution, the spectrophotometer was purged with air. Once the system reached a stable state, the absorbance was recorded at 412 nm to establish a baseline value. Subsequently, the absorbance of the ACh was measured both at the beginning and 10 min later at a temperature of 25 °C within the cuvette, which contained 5.21 of ACh. The quantification of AChE activity was conducted in microgram units (U/mg).

#### Neurotransmitters and its metabolites

Neurochemical analyses were conducted to assess the concentrations of several key neurotransmitters within the striatum. Utilizing high-performance liquid chromatography (HPLC) methodology, levels of ACh, DA, NE, 5-HT, GABA, and GLU were quantified. Striatal tissue homogenates were prepared and subjected to HPLC analysis. Neurotransmitter concentrations are reported in picograms per milligram (pg/mg)^24^.

#### Mitochondrial respiratory chain enzyme activities

##### Estimation of complexv (ATP)

Tissue homogenates were subjected to immediate sonication in an ice-cold 0.1 N perchloric acid solution to promptly inhibit ATPase activity. Subsequent centrifugation at 14,000 × g for 5 min at 4 °C facilitated the separation of cellular debris. The resulting supernatants were carefully neutralized using 1 N NaOH and subsequently stored at -80 °C for long-term preservation. Quantitative analysis of ATP concentrations within the supernatants was achieved through the utilization of RP-HPLC. The chromatographic separation was conducted using a 100 mM KH2PO4 buffer (pH 6.0) as the mobile phase, with a 1.2 mL/min flow rate. The chromatographic column was maintained at a constant temperature of 25 °C, and subsequently, the analyte was detected at 254 nm. A standard solution of ATP was meticulously prepared, adhering to established protocols^25^.

##### Estimation of complex II (SDH)

SDH activity serves as a marker for mitochondrial dysfunction within the brain. The assay quantifies the enzymatic conversion of succinate to fumarate by SDH in the presence of potassium ferricyanide as an electron acceptor. The methodology follows the protocol established by Sidhu et al. Briefly, the assay combines a sodium succinate solution with a brain homogenate fraction. Following incubation at 37 °C, p-iodonitrotetrazolium violet is introduced for further incubation. The reaction is terminated with a mixture of ethyl acetate, ethanol, and trichloroacetic acid. After centrifugation, absorbance is measured at 490 nm using a UV spectrophotometer. SDH activity is calculated using the chromophore’s molar extinction coefficient and presented as a percentage of control^26^.

#### Proinflammatory cytokines

To quantify the concentration of different inflammatory mediators (IL-1β, IL-6, TNF-α, and NF-κB), within brain tissue homogenates, ELISA was employed. Standard curves were utilized to determine the concentration of each marker, expressed in picograms per milliliter (pg/mL) of protein. All ELISA assays were performed meticulously, adhering to the protocols outlined by the respective kit manufacturers.

#### Endogenous antioxidants

The striatal tissue was carefully dissected from the brain for subsequent analysis. To assess the concentration of antioxidants, such as glutathione (GSH), superoxide dismutase (SOD), and catalase (CAT), were measured as previously reported methods^27^.

#### Nitrative and lipid peroxidation marker

Lipid peroxidation was determined in brain tissue homogenates, assessed as malondialdehyde (MDA) levels using the Wills et al. method^28^. Briefly, homogenates were incubated with thiobarbituric acid (TBA) at 90 °C for 10 min, followed by centrifugation. MDA concentration was quantified spectrophotometrically at 530 nm.

Nitric oxide (NO) production was evaluated by measuring nitrite levels using the Griess reaction. Briefly, nitrite in the homogenate reacted with the Griess reagent, forming a purple azo compound. The absorbance of this compound was quantified at 546 nm and was presented as µmol/g^29^.

#### Apoptotic and BDNF markers

Caspase-3, Caspase-9, and BDNF concentrations, serving as apoptosis markers, were measured utilizing commercially available ELISA kits. The ELISA procedures were meticulously assessed in adherence to the manufacturer’s established protocols to ensure consistent and accurate quantification of these inflammatory mediators. The resulting concentrations of these apoptotic markers were expressed in nanograms per milliliter (ng/mL).

### Histopathology of the brain

Brain tissue specimens were collected and underwent fixation in a 10% formalin solution. Subsequent to fixation, the tissue was embedded in paraffin. Paraffin blocks were sectioned at a thickness of 5 μm. The sections were then subjected to hematoxylin and eosin (H&E) staining. Histopathological examination was conducted on the stained sections using a bright-field microscope (Olympus, Japan) at a magnification of 200X to assess morphological alterations.

### Statistical analysis

Descriptive statistics of the data were demonstrated as mean ± SEM and visualized using GraphPad Prism version 8. Shapiro-Wilk tests were used to determine the normality of numerical variables. One-way analysis of variance (ANOVA) was utilized to evaluate statistically significant inter-group variations. Subsequent to ANOVA, Tukey’s post-hoc test was conducted to identify multiple comparisons. Statistical significance was considered at p-value < 0.05.

## Results

### Behavioural paradigms

#### Grip strength

**Fig. 3:**
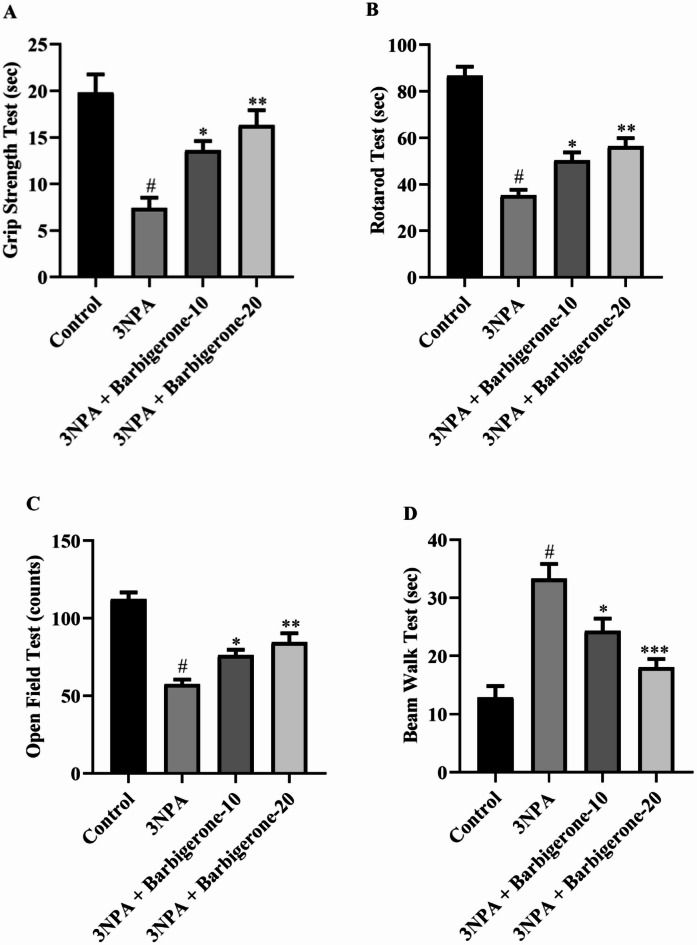
(A-D) Outcome of barbigerone on the behavioral assessment in HD rats. (A) Grip strength, (B) rotarod test, (C) open-field tests, (D) narrow beam walk test. Data are presented as mean ± S.E.M. (*n* = 6). A one-way ANOVA followed by Tukey’s post hoc test. #*P* < 0.001 vs. control; *P < 0.05, **P < 0.01, ***P < 0.001 vs. 3-NPA.

Figure 3A illustrates the effect of barbigerone on motor function in a HD rat induced by 3-NPA. Compared to a healthy control group, rats administered 3-NPA exhibited a significant reduction in grip strength, as evidenced by a decrease in the duration of wire-hanging (*p* < 0.001). However, barbigerone (10 and 20 mg/kg) markedly ameliorated grip strength deficits in 3-NPA-treated rats, resulting in a marked elevation in wire-hanging time in contrast to the 3-NPA group (F (3, 20) = 12.63, *P* < 0.0001).

#### Rotarod test

Motor function and coordination were assessed in rats using a rotarod apparatus, as depicted in Fig. 3B. The present study observed that rats showed a clear reduction in the duration spent holding the wire and the fall-off latency after receiving a 3-NPA injection compared with the control group. Furthermore, barbigerone treatment reduces motor abnormalities as evidenced by a significantly elevated total distance traveled as compared with the normal control group [F (3, 20) = 40.66, *P* < 0.0001].

#### Effect on open field strength

Figure 3C demonstrates the outcome of barbigerone on spontaneous locomotor activity in rats assessed via open-field tests. The data reveal a significant reduction in box crossings among rats treated with 3-NPA. However, barbigerone (10 mg/kg and 20 mg/kg) treatment significantly restored locomotor activity [F (3, 20) = 29.25, *P* < 0.0001]. These outcomes indicate that barbigerone therapy effectively ameliorates the locomotor deficits induced by 3-NPA in rats.

#### Narrow beam walking test

Figure 3D illustrates the outcome of barbigerone on narrow beam walk performance in rats. Compared to saline controls, 3-NPA-treated rats exhibited significantly prolonged traversal times (*p* < 0.0001) to cross wooden decks. High-dose barbigerone (20 mg/kg) significantly attenuated this deficit, reducing traversal times to levels comparable to 3-NPA. Low-dose barbigerone (10 mg/kg) also produced a significant, though less pronounced, improvement in traversal times when administered in conjunction with 3-NPA [F (3, 20) = 18.37, *P* < 0.0001].

### Biochemical analysis

#### Acetylcholinesterase

**Fig. 4:**
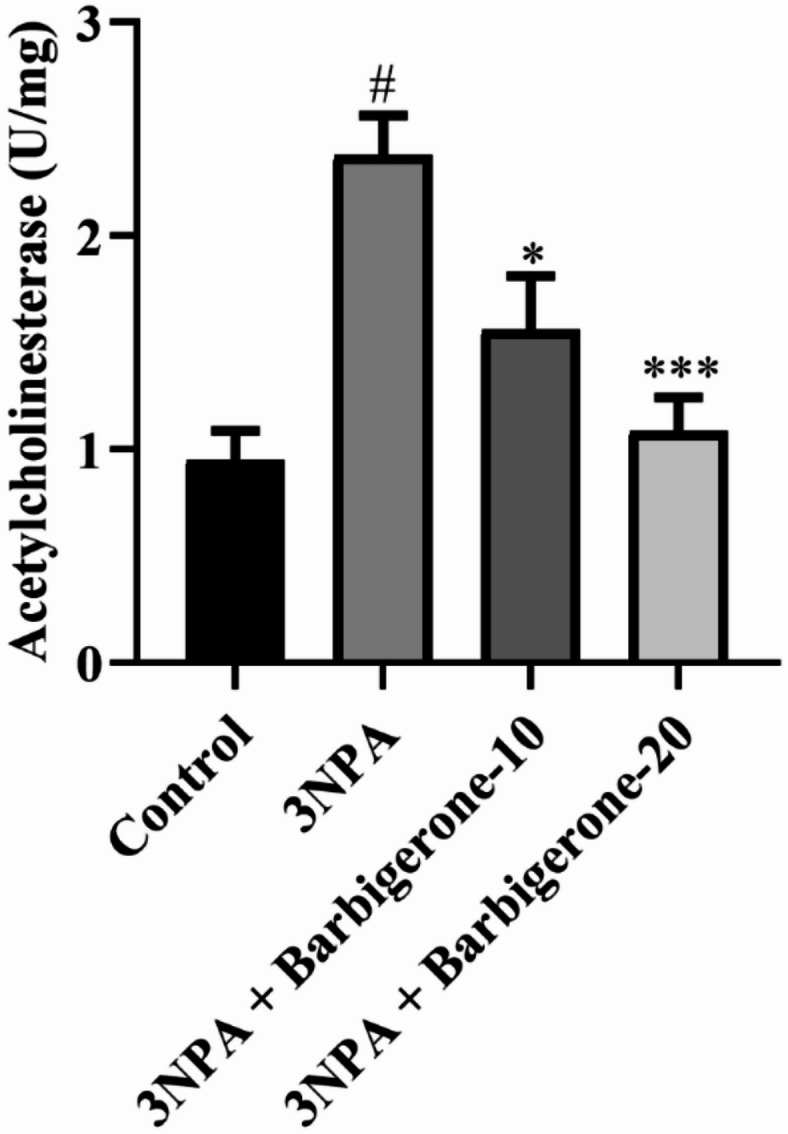
Outcome of the barbigerone on the level of AChE in rats. Results are expressed as mean ± S.E.M. (*n* = 6). A one-way ANOVA followed by Tukey’s post hoc test. #*P* < 0.001 vs. control; *P < 0.05, ***P < 0.001 vs. 3-NPA.

Figure 4 represents the effect of 3-NPA on AChE activity in rats. A significant elevation in AChE activity was found in the 3-NPA-induced group compared with the control (*P* < 0.001). Subsequent administration of barbigerone (10 mg/kg and 20 mg/kg) resulted in statistically reduced AChE levels compared with the 3-NPA group [F (3, 20) = 11.82, *P* = 0.0001].

#### Effect on neurotransmitter levels

**Fig. 5:**
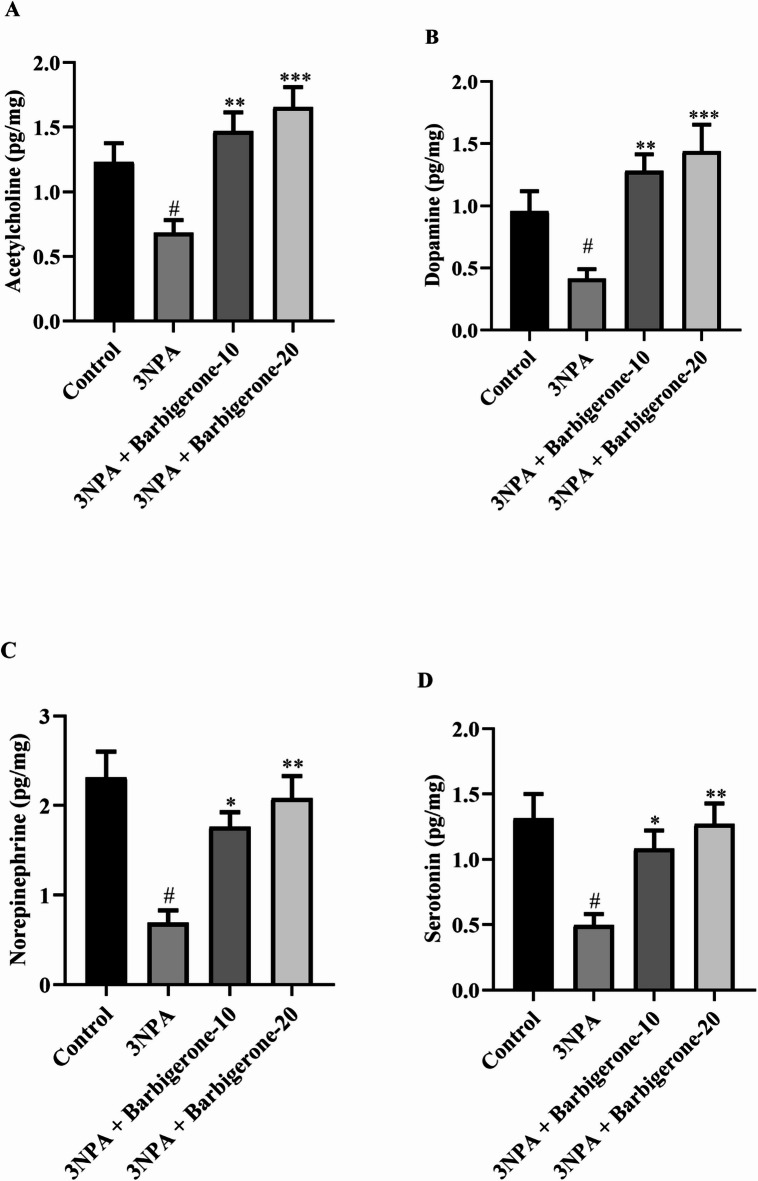

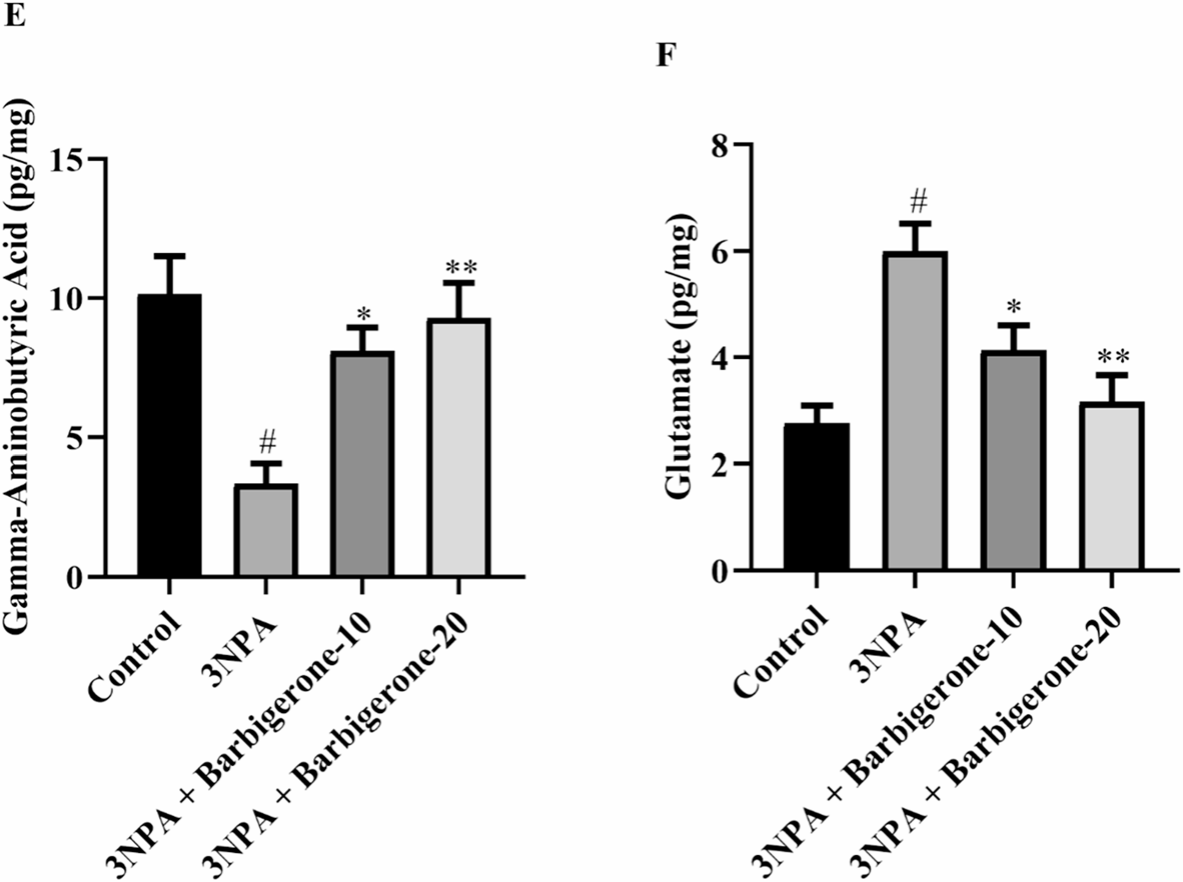
(A-F) Outcome of barbigerone on neurotransmitter (A) ACh, (B) DA, (C) NE, (D) 5-HT, (E) GABA, and (F) GLU levels. Values are expressed in mean ± S.E.M. (*n* = 6). A one-way ANOVA followed by Tukey’s post hoc test. #*P* < 0.001 vs. control; *P < 0.05, **P < 0.01, ***P < 0.001 vs. 3-NPA.

Figure 5 (A-F) illustrates the neurotransmitter alterations observed in a rat with HD evoked by 3-NPA. 3-NPA caused a marked depletion of ACh, DA, NE, 5-HT, and GABA, while concurrently elevated GLU levels compared to the control group. Notably, treatment with barbigerone at both 10 and 20 mg/kg significantly corrected these neurotransmitter imbalances. Barbigerone administration effectively increased the ACh [F (3, 20) = 9.301, *P* = 0.0005], DA [F (3, 20) = 8.438, *P* = 0.0008], NE [F (3, 20) = 10.80, *P* = 0.0002], 5-HT [F (3, 20) = 6.493, *P* = 0.0030], and GABA [F (3, 20) = 7.839, *P* = 0.0012], while simultaneously attenuating the elevated GLU [F (3, 20) = 9.511, *P* = 0.0004] levels compared to the 3-NPA group.

#### Mitochondrial respiratory chain enzyme activities

**Fig. 6:**
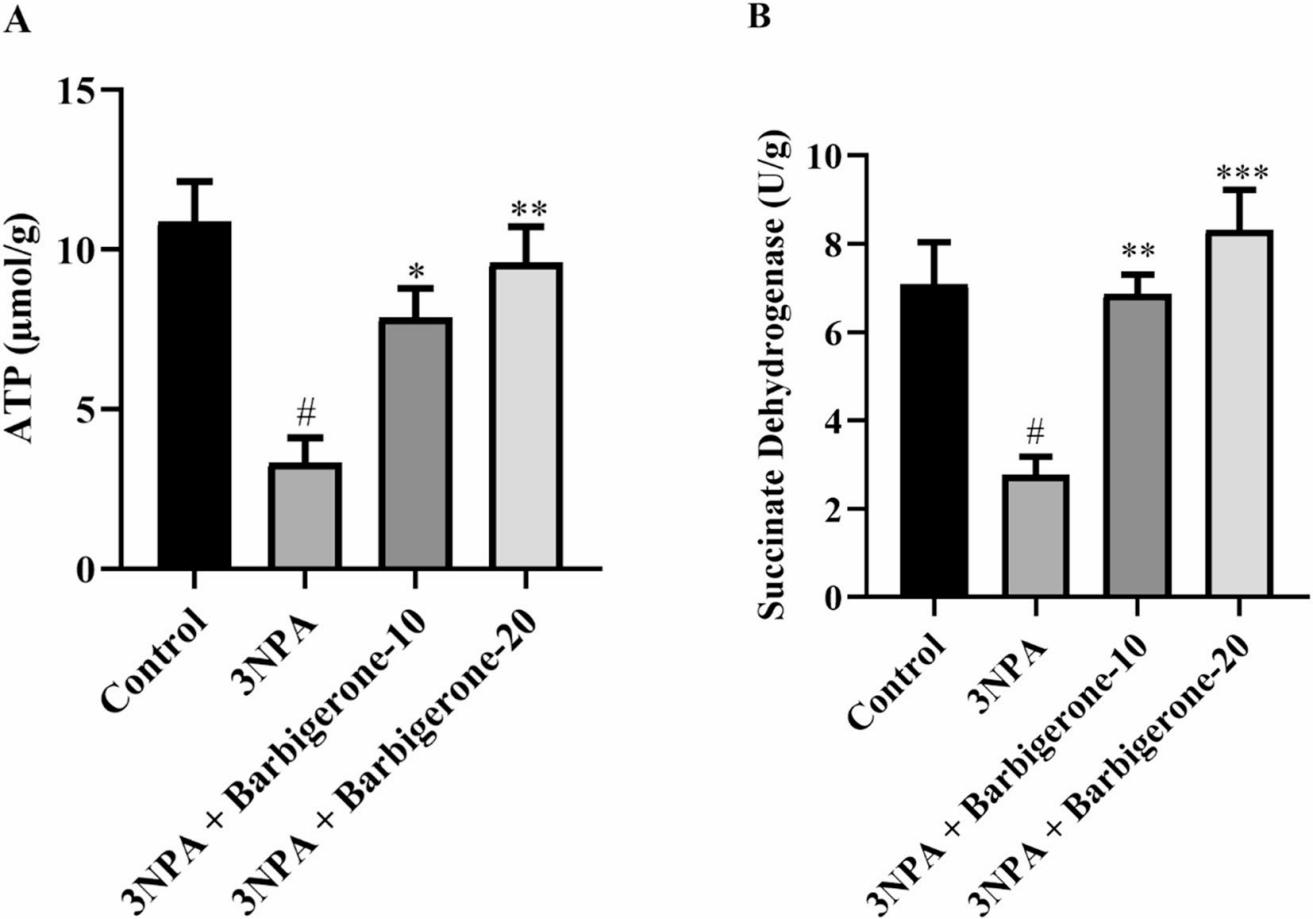
(A-B) Outcome of barbigerone on mitochondrial respiratory chain enzyme activity, as assessed by (A) ATP and (B) SDH levels. Results are expressed as mean ± S.E.M. (*n* = 6). A one-way ANOVA followed by Tukey’s post hoc test. #*P* < 0.001 vs. control; *P < 0.05, **P < 0.01, ***P < 0.001 vs. 3-NPA.

Figure 6(A-B) illustrates the outcome of barbigerone on mitochondrial respiratory chain enzyme activity, as assessed by ATP and SDH levels. 3-NPA treatment significantly decreased both ATP and SDH levels compared to the control group. Conversely, administration of barbigerone at both doses markedly increased ATP [F (3, 20) = 10.20, *P* = 0.0003] and SDH [F (3, 20) = 11.08, *P* = 0.0002] levels compared to the 3-NPA group.

#### Estimation of proinflammatory cytokines

**Fig. 7:**
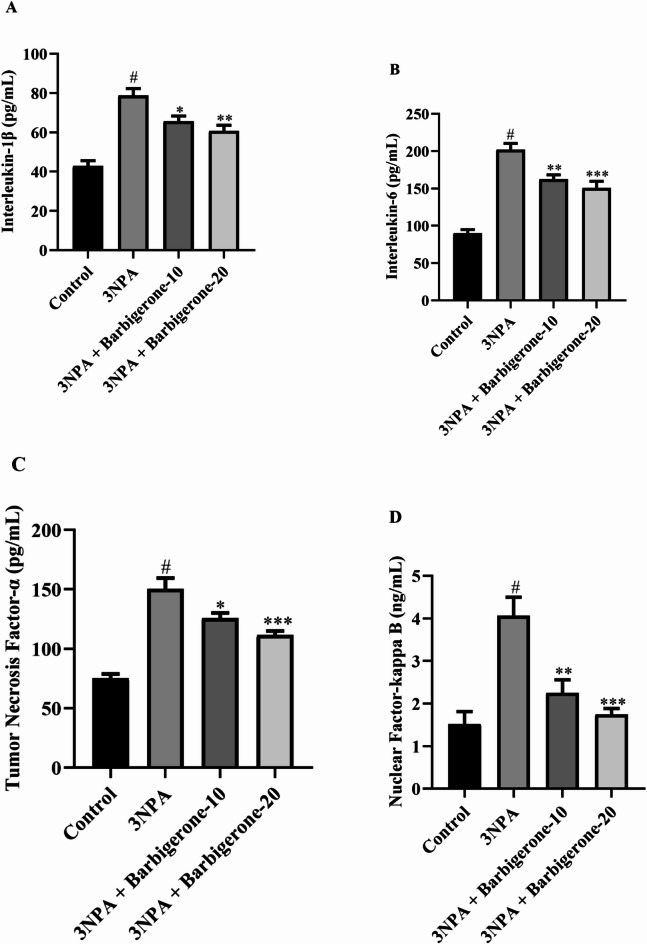
(A-D) Outcome of barbigerone in the regulation of proinflammatory indicators (A) IL-1β, (B) IL-6, (C) TNF-α, and (D) NF-κB. Values are expressed in mean ± S.E.M. (*n* = 6). A one-way ANOVA followed by Tukey’s post hoc test. #*P* < 0.001 vs. control; *P < 0.05, **P < 0.01, ***P < 0.001 vs. 3-NPA.

Figure 7 (A-D) illustrates the modulatory effect of barbigerone on endogenous proinflammatory mediators. Compared to control, the 3-NPA-treated rats showed a significant elevation of proinflammatory markers such as IL-1β, IL-6, TNF-α, and NF-κB (*P* < 0.05). Barbigerone treatment at doses of 10 mg/kg and 20 mg/kg showed statistically significant reduction in the TNF-α [F (3, 20) = 30.63, *P* < 0.0001], IL-1β [F (3, 20) = 24.84, *P* < 0.0001], IL-6 [F (3, 20) = 40.24, *P* < 0.0001], and NF-κB [F (3, 20) = 13.54, *P* < 0.0001] compared to the 3-NPA group.

#### Endogenous antioxidants

**Fig. 8:**
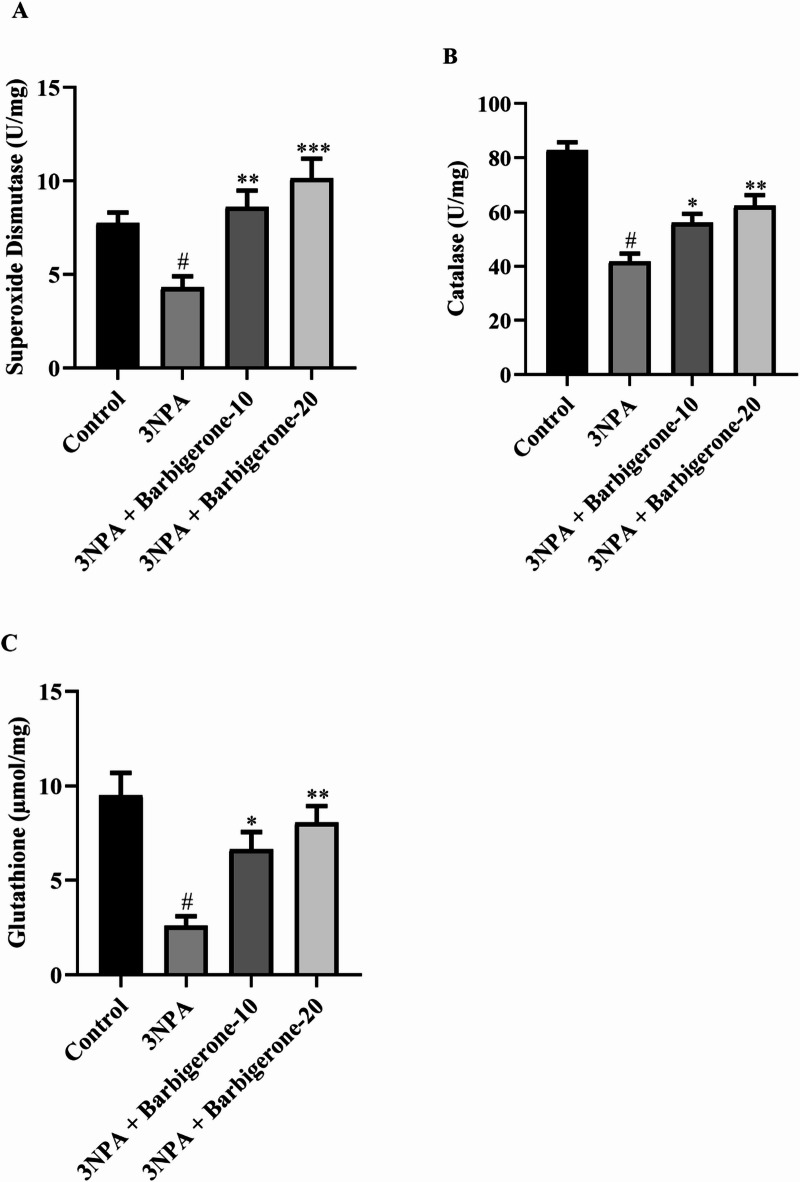
(A–C) Outcome of barbigerone on the various antioxidant enzymes such as (A) SOD, (B) CAT, and (C) GSH. Results are expressed as mean ± S.E.M. (*n* = 6). A one-way ANOVA followed by Tukey’s post hoc test. #*P* < 0.001 vs. control; *P < 0.05, **P < 0.01, ***P < 0.001 vs. 3-NPA.

Figure 8 (A-C) illustrates the impact of barbigerone on endogenous antioxidant enzyme activities, including SOD, CAT, and GSH. Compared to the 3-NPA group, which exhibited significant reductions in SOD, CAT, and GSH levels (*P* < 0.05), barbigerone at doses of 10 and 20 mg/kg significantly increased SOD [F (3, 20) = 9.672, *P* = 0.0004], CAT [F (3, 20) = 27.54, *P* < 0.0001], and GSH [F (3, 20) = 10.84, *P* = 0.0002] levels, respectively.

#### Nitrative and lipid peroxidation marker

**Fig. 9:**
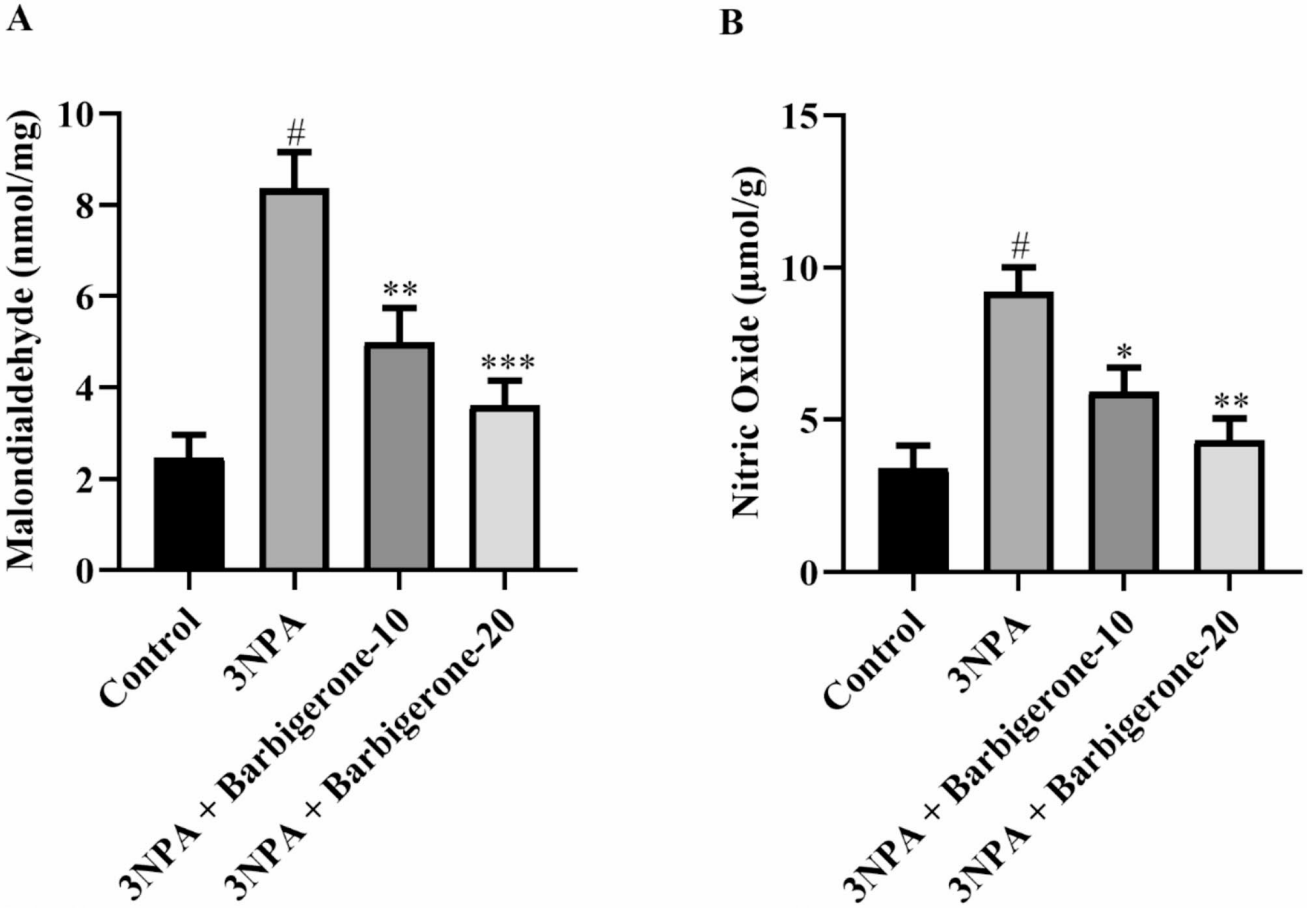
(A-B) The impact of barbigerone on markers of (A) MDA and (B) NO. Values are expressed in mean ± S.E.M. (*n* = 6). A one-way ANOVA followed by Tukey’s post hoc test. #*P* < 0.001 vs. control; *P < 0.05, **P < 0.01, ***P < 0.001 vs. 3-NPA.

Figure 9 (A-B) illustrates the impact of barbigerone on markers of nitrative and lipid peroxidation. The investigation revealed that 3NP administration increased MDA and NO levels compared with the control group, indicative of oxidative and nitrative stress. Conversely, treatment with barbigerone at both doses resulted in a notable reduction in MDA [F (3, 20) = 14.93, *P* < 0.0001] and NO [F (3, 20) = 10.92, *P* = 0.0002] levels compared to the 3-NPA group.

#### Apoptotic and BDNF markers

**Fig. 10:**
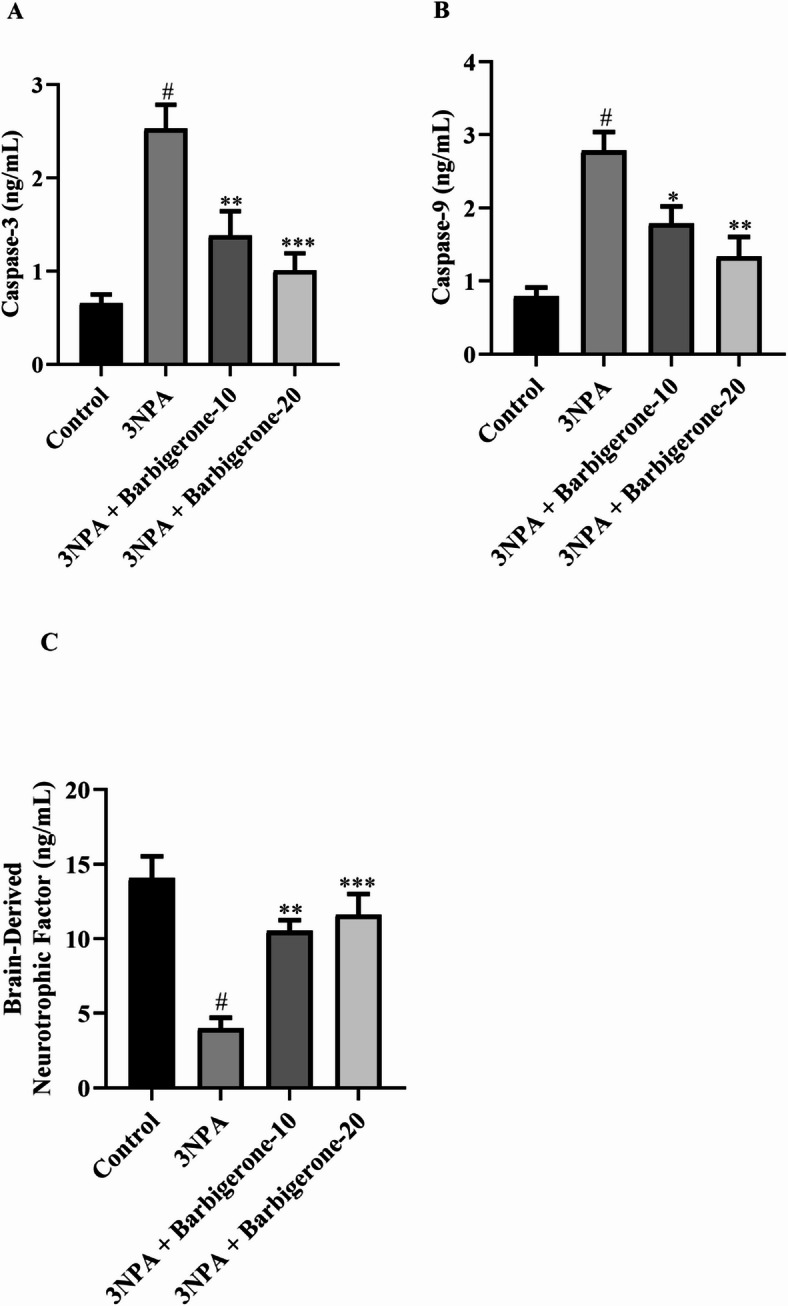
(A-C) Outcome of barbigerone on key apoptotic and BDNF markers, namely (A) Caspase-3, (B) Caspase-9, and (C) BDNF. Values are expressed in mean ± S.E.M. (*n* = 6). A one-way ANOVA followed by Tukey’s post hoc test. #*P* < 0.001 vs. control; *P < 0.05, **P < 0.01, ***P < 0.001 vs. 3-NPA.

Figure 10 (A–C) demonstrates the modulatory effects of barbigerone on markers, namely BDNF, Caspase-3, and Caspase-9, in 3-NPA-induced HD. 3-NPA administration markedly elevated Caspase-3 and Caspase-9, indicative of increased apoptotic activity, while concurrently leading to a reduction of BDNF, a neurotrophic factor critical for neuronal survival. Treatment with both 10 mg/kg and 20 mg/kg doses of barbigerone significantly attenuated these 3-NPA-induced alterations. Specifically, barbigerone administration markedly reduced the levels of both Caspase-3 [F (3, 20) = 15.19, *P* < 0.0001] and Caspase-9 [F (3, 20) = 13.83, *P* < 0.0001], while concurrently increased the levels of BDNF [F (3, 20) = 14.64, *P* < 0.0001] compared to the 3-NPA group.

### Histopathology

**Fig. 11:**
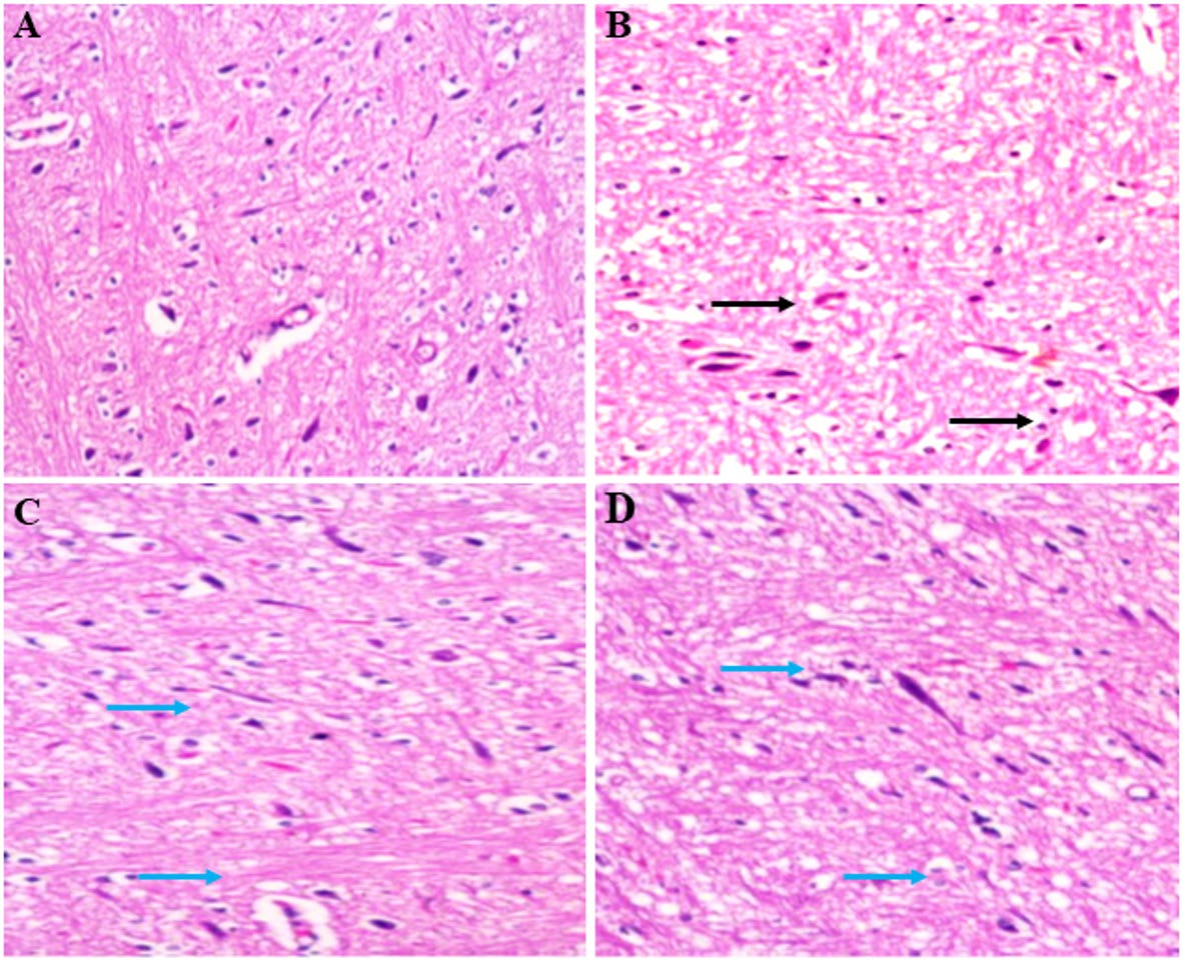
(A-D) Histopathological examination of the striatum showing the effect of barbigerone on 3-NP-induced HD rat models. The black arrow shows severe degeneration in the striatum, and the blue arrows show moderate to mild degeneration in the striatum.

Figure 11 (A-D) represents the histopathological examination of the striatum in 3-NPA-induced HD rat models, which was conducted to assess the effect of barbigerone. Control groups exhibited a normal striatal architecture characterized by uniformly arranged neurons with basophilic cytoplasm, vesicular nuclei, and distinct perineuronal spaces. In contrast, the 3-NPA group demonstrated histopathological alterations, including nuclear pyknosis, gliosis, satellitosis, and neuronophagia in striatal neurons. Administration of both doses of barbigerone effectively reduced these histopathological changes and improved the cellular structure of the striatum.

## Discussion

HD is an autosomal dominant neurodegenerative disorder that leads to progressive motor dysfunction, cognitive decline, and psychiatric disturbances, primarily due to the expansion of CAG repeats in the HTT gene, resulting in mutant huntingtin protein aggregation^3^. The 3-NPA rat model replicates several hallmarks of HD pathology, including striatal degeneration, mitochondrial dysfunction, oxidative stress, and motor abnormalities, making it a well-established and reliable model for preclinical studies^30^. 3-NPA is a mitochondrial toxin that irreversibly inhibits SDH, a key enzyme in the tricarboxylic acid (TCA) cycle and electron transport chain, thereby impairing ATP production and inducing oxidative stress^31^. Systemic administration of 3-NPA has been shown to induce HD-like symptoms such as weight loss, cognitive impairment, and motor abnormalities, including impaired balance, reduced grip strength, and diminished locomotor coordination, along with biochemical changes that mirror the disease progression observed in patients^32^.

In the present study, administration of 3-NP (10, 20 mg/kg, i.p., for 14 days) produced a significant decrease in body weight, motor and cognition-related behaviors and increased oxidative stress in the brain, well supporting the above findings. In line with previous literature, 3-NPA administration led to pronounced impairments in motor function, decreased grip strength, rotarod performance, open-field activity, and increased traversal time on the narrow beam test, reflecting HD-like motor and coordination deficits^33^. This was associated with mitochondrial dysfunction, leading to disruptions in the neurotransmitter systems crucial for motor control and muscle activity^22^. Barbigerone treatment significantly improved motor deficits associated with HD, demonstrating its motor-protective potential. Treated rats showed enhanced grip strength, indicating improved muscle function, and performed better on the rotarod, reflecting improved coordination and balance. In the open field test, 3-NPA reduced locomotor activity, while barbigerone increased exploration, total distance travelled, and time spent in the arena, suggesting reduced anxiety and improved motor function. These findings are consistent with the outcomes of prior investigations^19,34^.

Neurotransmitter dysregulation constitutes a pivotal factor in the manifestation of both motor and cognitive deficits observed in HD. Cognitive impairments, particularly memory dysfunction, are primarily attributed to an imbalanced neurotransmitter milieu within the cholinergic nervous system of HD patients. This is evidenced by elevated levels of AChE, an enzyme responsible for ACh degradation. Notably, the loss of cholinergic innervation, a hallmark of numerous neurodegenerative disorders, is associated with increased AChE activity. Conversely, AChE inhibition leads to prolonged ACh availability, thereby enhancing cholinergic function. The present study demonstrates a significant elevation in AChE activity in the 3-NPA-induced group compared with the control group. However, both doses of barbigerone demonstrated a significant reduction in AChE activity. This outcome suggests that barbigerone has the potential to modulate AChE activity, which is often dysregulated in neurodegenerative disorders, including HD. The observed significant elevation in AChE activity in the 3-NPA-evoked rats compared with the control group aligns with prior research and reflects the neurotoxic effects induced by 3-NPA^34^. Furthermore, DA, GABA, and Glu have emerged as significant contributors to the pathophysiology of HD. Moreover, ACh, DA and 5HT, which play crucial roles in regulating movement, emotion, and other essential functions, exhibit altered levels in individuals with HD^35^. Moreover, the present neurotransmitter results revealed altered levels of ACh, DA, NE, 5-HT, GABA, and GLU in the 3-NPA group. DA and 5-HT imbalances contribute to both motor dysfunction and psychiatric symptoms, while reduced GABA and GLU levels reflect disrupted inhibitory-excitatory signaling due to striatal degeneration. NE alterations have been associated with impaired attention and increased anxiety in HD^7,36^. Barbigerone treatment restored these neurotransmitter levels, confirming its broad-spectrum neuromodulatory potential. These findings indicate that barbigerone not only mitigates cholinergic dysfunction but also acts broadly across multiple neurotransmitter systems implicated in HD pathophysiology, thereby enhancing motor, cognitive, and emotional outcomes.

There is extensive evidence for bioenergetic deficits and mitochondrial dysfunction in HD. Mitochondria, crucial for neuronal function, play vital roles in energy metabolism, calcium homeostasis, and signal transduction. As the primary site of oxidative phosphorylation, they are responsible for generating ATP, the cellular energy currency. Furthermore, mitochondria are integral to apoptotic pathways and cellular redox regulation. Mitochondrial dysfunction has been associated with the pathogenesis of several diseases. SDH, a key enzyme, serves as a valuable biomarker for assessing mitochondrial function and evaluating the efficacy of neuroprotective agents against 3-NPA. By inhibiting SDH, 3-NPA induces energy metabolic impairments and oxidative stress, making it a widely accepted model for studying mitochondrial dysfunction in neurodegenerative diseases^37^. The present investigation showed a marked reduction in both ATP and SDH levels in the 3-NPA-treated group compared with the control group. Conversely, the administration of barbigerone at both dosages markedly elevates the ATP and SDH levels. These findings are in alignment with the results of a previous study^37,38^.

Elevated levels of proinflammatory cytokines, including IL-1β, IL-6, TNF-α, and those regulated by NF-κB, can induce neurotoxicity and impaired synaptic plasticity. Synaptic plasticity is a fundamental mechanism underlying learning and memory. Consequently, excessive cytokine production can contribute to cognitive impairment^39–41^. The administration of 3-NPA induced neurotoxicity, triggering the pro-inflammatory responses. The elevated levels of IL-1β, IL-6, TNF-α, and NF-κB exacerbate neuronal damage. The remarkable reduction in IL-1β, IL-6, TNF-α, and NF-κB levels following barbigerone administration (10 and 20 mg/kg) suggests a potential anti-inflammatory effect of barbigerone. This anti-inflammatory effect of barbigerone could contribute to the amelioration of neurodegenerative processes and align with the previously reported study^42^.

Oxidative stress contributes to neuronal death in HD by disrupting redox homeostasis. Oxidative stress has been proposed to play a significant role in the pathogenic mechanism of HD^43^. 3-NPA, a toxin known to inhibit SDH in the mitochondria, induces energy impairment and oxidative stress. 3-NPA administration has been shown to increase MDA and NO levels, while concomitantly depleting GSH, SOD, and CAT in brain tissue^37,44^. The present investigation observed a significant reduction of GSH, SOD, and CAT, alongside an elevation of MDA, and NO, within the 3-NPA control group. These findings collectively suggest a lowered reactive oxygen species (ROS) antioxidant defense system within this group, highlighting the pivotal role of these enzymes in scavenging ROS and shielding cells from oxidative damage. Conversely, treatment with barbigerone, at both doses, resulted in a marked augmentation of GSH, SOD, and CAT activities, concomitantly with a significant attenuation in MDA and NO levels. These observations strongly indicate the potential of barbigerone to enhance endogenous enzymatic antioxidant defenses. This study suggests that barbigerone may contribute to the restoration of redox homeostasis and provide enhanced cellular protection against oxidative stress, corroborating findings from previous study^37^.

Neurodegeneration is intricately linked to neuroapoptosis, a cellular process characterized by programmed cell death. This pathological process is often exacerbated by a confluence of factors, including oxidative stress, mitochondrial dysfunction, excessive calcium influx, and the overproduction of ROS^19^. BDNF, a crucial member of the neurotrophin family, is synthesized in the cortex and subsequently transported to the striatum. Within the striatum, BDNF serves a crucial role in neuronal function and development. The administration of 3-NPA was observed to amplify the apoptotic cascade within the striatum. This was evidenced by an upregulation of apoptotic proteins, specifically caspase-3 and caspase-9, along with a concomitant downregulation of the neurotrophic factor BDNF^19,45^. Our study demonstrates that 3-NPA administration resulted in a significant elevation of Caspase-3 and Caspase-9, indicative of increased apoptotic activity, while concurrently leading to a reduction of BDNF, a neurotrophic factor critical for neuronal survival. Administration of barbigerone markedly attenuated these 3-NPA-induced alterations. Specifically, barbigerone treatment significantly reduced both Caspase-3 and Caspase-9, while concurrently increasing the levels of BDNF. These findings suggest that barbigerone exerts its neuroprotective effects^45^.

**Fig. 12:**
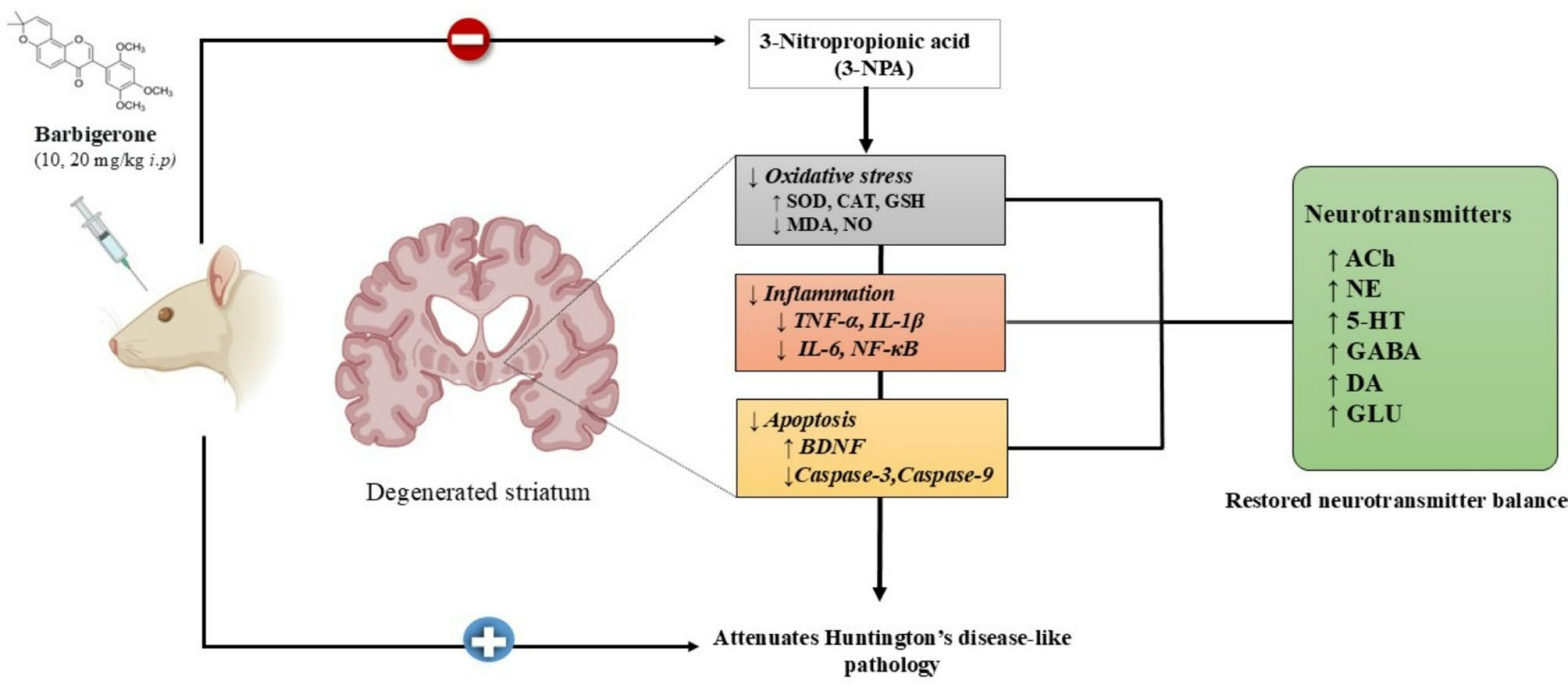
Proposed mechanism of barbigerone on 3-NP-induced rats.

Histopathological analysis further revealed that 3-NPA induced characteristic striatal damage, including neuronal loss, gliosis, and pyknosis, indicative of excitotoxicity and mitochondrial dysfunction. Barbigerone treatment (10 and 20 mg/kg) markedly preserved striatal architecture, with reduced neuropathological alterations and intact neuronal morphology, supporting its neuroprotective effect in HD.It is hypothesized that barbigerone exerts its neuroprotective effects in 3-NPA-induced HD through a multi-targeted mechanism involving modulation of neurotransmitters (ACh, DA, 5-HT, GABA, Glu), inhibition of AChE, enhancement of endogenous antioxidant defenses (GSH, SOD, CAT), preservation of mitochondrial function (ATP and SDH), suppression of proinflammatory cytokines (IL-1β, IL-6, TNF-α, NF-κB), and attenuation of apoptosis via caspase-3/-9 inhibition and BDNF upregulation. These integrated actions collectively contribute to the mitigation of neurodegeneration and improvement of motor and cognitive functions in HD (Fig. 12).

This study demonstrates the potential neuroprotective effects of barbigerone against 3-NPA-induced HD in rats; however, it has certain limitations. The short duration and limited animals used in behavioral assessments, and the restricted evaluation of cytokine markers poses significant constraints. The limited sample size may compromise the robustness and generalizability of the findings due to potential impacts on statistical power and increased susceptibility to outliers. Increasing the sample size could enhance statistical power, minimize the influence of outliers, and improve the precision of conclusions.

Notably, this is the first study to demonstrate the multitargeted neuroprotective effects of barbigerone in a 3-NPA-induced HD rat model, involving neurotransmitter modulation, mitochondrial restoration, redox balancing, anti-inflammatory action, and apoptosis inhibition.

To further elucidate the mechanisms underlying barbigerone’s neuroprotective effects, further investigations are necessary. Future research should incorporate techniques such as immunohistochemistry, Western blotting, and reverse transcription-polymerase chain reaction (RT-PCR) to delve into molecular and cellular pathways. Moreover, expanding the scope of research to include tissue immunohistochemistry, utilizing alternative animal models, and exploring gene and protein expression patterns would provide a more comprehensive understanding of barbigerone’s therapeutic potential.

## Conclusion

The potential therapeutic effect of barbigerone in HD rats induced by 3-NPA showed mitigation in motor deficits, enhancing motor coordination, muscle strength, and exploratory behavior. It demonstrated positive effects on biochemical markers, including the modulation of AChE activity, reduction in oxidative stress, and restoration of endogenous antioxidants. Furthermore, barbigerone exhibited anti-inflammatory properties by decreasing proinflammatory cytokine levels and positively influenced neurotransmitter levels, critical for cognitive function. Histopathological assessments supported its potential neuroprotective effects. The findings strongly suggest that barbigerone holds a remarkable outcome as a potential treatment for neurodegenerative conditions like HD.

## Data Availability

The authors declare that the data supporting the findings of this study are available within the paper. Should any raw data files be needed in another format, they are available from the corresponding author upon reasonable request.
